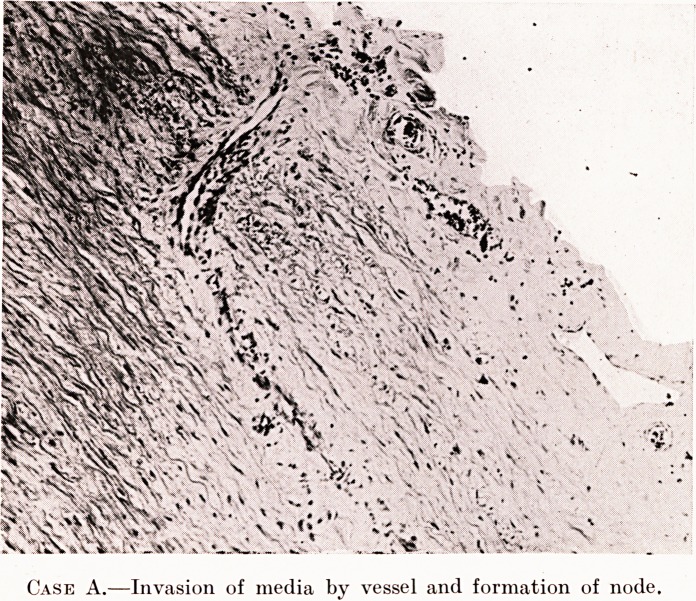# The Histology of the Aortic Wall in Acute Rheumatism
*This Essay was awarded the Martyn Memorial Pathological Scholarship for 1929.From the University Centre of Cardiac Research, General Hospital, Bristol.


**Published:** 1929

**Authors:** J. J. Giraldi


### THE HISTOLOGY OF THE AORTIC WALL IN ACUTE RHEUMATISM.*

BY

J. J. GIRALDI.

THE histology of the aortic wall in acute rheumatic infection has until recent years attracted very little attention, especially in this country. Recently much work has been devoted to the subject of acute rheumatism, and the aorta has received its due share, chiefly at the hands of American observers.

In the literature the changes in the aorta have usually been classified as an acute aortitis, and rheumatic fever has been ranged with those diseases giving rise to a similar condition, viz. the exanthemata, gonorrhœa, acute tuberculosis, etc. (Allbutt[1]). As far back as 1912 Klotz[2] drew attention to the occurrence of unusual changes in the media. He gives an account of three necropsies of consecutive cases of rheumatic fever in young patients. The intima was not altered, but the media was thickened by increase of vascular elements, œdema and perivascular infiltration, with abundance of leucocytes and plasma cells. He noted signs of fibrous repair, and mentioned the possibility of this change predisposing to aneurysm. Coombs[3] in 1908 notes the presence of whitish-yellow patches at

* This Essay was awarded the Martyn Memorial Pathological Scholarship for 1929.

From the University Centre of Cardiac Research, General Hospital, Bristol.

the root of the aorta in rheumatic carditis, which proved inflammatory in nature.

This paper is an attempt to describe the appearances observed in five consecutive cases of rheumatic fever. One case was studied in some detail and forms the basis of this description.*

Briefly, the cases are as follows:—

(A) F. G., boy, aged 13 years. Rheumatic fever six years before death; since then acute bouts. Died in acute exacerbation. *P.M.*—Chronic and acute mitral and aortic endocarditis, adherent pericardium, hypertrophy and dilatation of all chambers of the heart.

(B) W. L., man, aged 60 years. Brought to hospital *in extremis;* died next day—little history obtainable. *P.M.*—Malignant disease of root of lung. Old and recent valvular disease of aortic and mitral valves. Adherent pericardium. Chronic interstitial nephritis.

(C) Boy, aged 5 years. Died in acute exacerbation of carditis. Heart lesion of about six months' standing. *P.M.*—Recent endocarditis of mitral and aortic valves. Adherent pericardium.

(D) J. C., girl, aged 7 years. Heart lesion of two to three years' duration. Died suddenly with acute carditis. *P.M.*—Recent pericarditis, with acute and chronic mitral mural endocarditis.

(E) A. W., girl, aged 14½ years. History of rheumatic fever of three years' duration. Died suddenly with acute carditis. *P.M.*—Recent pericarditis and mediastinitis, with recent endocarditis of mitral and aortic valves.

It will be noted that all five cases showed evidences post-mortem of recent infection.

*Macroscopically,* dilatation of the aorta was noted in two cases. The intima showed in four cases raised

* All the figures at the end of the Paper were taken from this case, but they may be regarded as representative of the changes found in all the cases.

whitish-yellow patches, some of them linear in outline, others irregular with crenated margins, never exceeding half a square centimetre. These patches are found almost invariably in the first half-inch or so of the aorta, usually in the neighbourhood of the aortic valves. In one case a thickened patch was present in the neighbourhood of the innominate artery ; similar patches, however, were seen at the root of the aorta. These streaks and patches bear a naked-eye resemblance to the so-called "fatty streaks" observed frequently in cases of typhoid, tuberculosis, septicæmia, etc. This resemblance is, however, only superficial, and on closer examination it will be observed that the rheumatic patches are whiter, less glistening and fatty-looking, and have a harder consistence if an attempt is made to scratch them with the finger. This was well shown in a case, not included in this paper, where both types of patches were present. In rheumatic cases these patches tend to be irregular in outline rather than streaky.

*Microscopically*, in all the sections examined the intima was found thickened. The thickening, though present throughout, is not uniform in extent ; at one spot the intima gradually thickens and forms a well-marked "hillock," bulging into the lumen of the aorta, an appearance somewhat resembling the endarteritis deformans of the smaller arteries.

The endothelial lining in places is destroyed, probably as an artefact. In places it appears swollen and, most conspicuous of all, there are signs of endothelial proliferation in many sections, the endothelial cells being heaped up on the outer side of the endothelium. In many sections there is a puckering, zigzag in outline, of the endothelial margin, usually most pronounced at the sides of the maximal intimal thickening. The intima, being probably œdematous before death, has shrunk in the process of preparation, the stretched endothelial lining now having to accommodate itself to the decreased superficies. On the whole, the endothelial picture is one of proliferation.

The intimal thickening is found to be composed of thick, wavy bands of collagenous material, running longitudinally as a rule, but in a few instances turning inwards or outwards. In places this degenerative matrix invades the media and is continuous with the intermuscular substance of the media, with the consequent destruction of the internal elastic layers. In this groundwork of the intima there are numerous open spaces, some large and irregular in outline, others small and rounded. As will be shown below, some of the smaller of these are fatty in nature, but the majority are not. Their true nature I have not found out. They may be due to fluid exudation. Attempts to demonstrate fibrin in them have not succeeded, but these have not been very exhaustive; moreover, a fluid exudate need not necessarily contain demonstrable fibrin.

There is considerable infiltration of the intima; the cells are mostly of endothelioid and lymphocytic type. The endothelial cells are in many cases present near the endothelium of the intima, from which they can be seen to be actively proliferating. The lymphoid are smaller, rounded, with a deeply-staining nucleus filling most of the cell-body, the latter staining faintly. The endothelioid cells are larger, of elongated shape

PLATE XVI.

Case A.—Aortic valves, with root of aorta. Shows some raised patches just above the valves.

Case A.—Typical focal lesion in media. Shows vessel with lymphocytes and a few large endothelioids.

### PLATE XVII.

Case A.—Orcein. Shows band of elastic to left of intimal "hillock," also absence at sites of medial lesions.

Case A.—Invasion of media by vessel and formation of node.

with blunt ends, and a pale nucleus with loosely-arranged chromatin.

I find the maximum cellular increase in the intimal "hillocks." Here they are mostly lymphocytes, but as we trace the intima round and it gradually decreases in thickness, the endothelioid cells come into the picture to the almost total exclusion of the lymphoid cells. All these cells are regularly distributed throughout the intima. In some cases the cells are placed in rows between the collagenous bands, forming the "palisade" appearance described by McCallum[4] in the auricular endocardium and by Pappenheimer and Von Glahn[5] in the aorta. This appearance has been the exception rather than the rule in the cases I have examined. This arrangement is due, no doubt, to the peculiar structure of the part, the collagenous bands when well formed pushing the cellular elements out of their course, so that they come to lie by their side. If the cells are too numerous this does not seem to occur. As has been mentioned above, when we trace the intimal "hillock" laterally we find that the spindle-shaped endothelial cells come to lie in rows parallel to the vessel wall. In sections stained with van Gieson there is well-formed fibrous tissue in this area. Some of the lymphocytes in all sections show signs of degeneration; their nuclei have lost their shape and become drawn and pyriform, while in some cases they are fragmented and lie free in the collagenous matrix. The endothelioid cells do not show such evident signs of degeneration; some of them possess two nuclei.

In some places the intima shows an appearance described by Pappenheimer and Von Glahn[5]; the cells lie with their drawn-out pyknotic nuclei at right angles to the endothelial surface of the vessel. At one spot, in a section, there is a dense collection of cells, so that it stands out from the rest. In this agglomeration of cells lymphocytes mostly predominate ; some in varying stages of degeneration. Some endothelials, round and elongated, are also present. In the centre of this cellular collection there are a few clear spaces. Just outside this lymphocytic zone, and on the outer aspects of the endothelium, there are a number of spindle-shaped cells, proliferating from the endothelial lining of the aorta, and making for this cellular zone ; some are already around it. This appearance suggests an inflammatory response of a subacute nature. With toluidine blue sections the basophilic affinities of these cells are well demonstrated. Methyl-pyronin green-stained sections show an occasional plasma-cell in the intima.

Some sections were stained for organisms, but none were discovered.

*Media and adventitia.*—Many sections show a looseness and loss of the normal compact arrangement of the medial elements in its inner half. In places the collagenous matrix of the media is directly continuous with that of the intima through gaps in the internal elastic layers. There is an increase of the intercellular matrix in the inner half of the media ; in some sections this appearance contrasts with the more compact outer half, whereas in other sections the whole width of the media presents this looseness in its architecture. In some cases the inner layers of the media are so altered that there is no abrupt transition into intima.

The muscle cells themselves vary in appearance; many of them appear healthy and take the stains well. Some, however, have lost their distinct cell outlines, nuclei have become less conspicuous, and in places there is a definite paucity of nuclei. The muscle cells are in cases thinned and atrophied as if compressed. In other sections the muscle nuclei, or in some cases fragments of them, lie scattered in the intercellular collagenous matrix. These changes are best marked under the greatest intimal thickening.

In the outer half of the media there is a different picture. The muscle cells and elastic fibres, in so far as they are present, appear normal; the compact arrangement is not grossly disturbed, and there is little, if any, increase of intermuscular substance.

Scattered throughout the outer third of the media there are areas of complete loss of the essential medial elements, muscle and elastic, and this is replaced by small focal lesions of an unusual character. These lesions present different appearances in outline and in histological structure. In some cases they are small and oval in outline, in others they consist of narrow bands lying circularly around the vessel wall. These appearances are no doubt determined by the histology of the part; as the branches of the vasa vasorum enter the media they invade it to a variable extent, usually to about its outer third only, and then divide into fine lateral branches which run circularly around the vessel; along these the lesions are apparently distributed. As they increase in size they are modified in their spread by the muscular and elastic fibres.

These foci differ in structure according to their age and probably to the virulence of the infection. A typical focus consists of a small, thin-walled blood-vessel, usually containing some red cells. This vessel may show endothelial proliferation. Around it there is a collection of cells of two types; some are of the usual lymphoid type, with a deeply-staining nucleus filling the cell-body. The other cells are less regular in shape; they may be round, but are usually large and fusiform with blunt ends. They have a pale, anæmic-looking nucleus with loosely-arranged chromatin, which gives it a characteristic vacuolated appearance. In a few cases two or three nuclei may be present in one cell, arranged in a row along the long axis of the cell. Toluidine blue brings out the chromatic arrangement very well and the vacuolated appearance is very evident. These cells correspond in appearance and staining characteristics with those described by Coombs[6] as occurring in the submiliary nodules in the myocardium in rheumatic fever.

The whole lies in a structureless collagenous matrix which may contain remnants of muscle and elastic tissue. This ground substance is not sharply demarcated; at its periphery it insinuates itself between the muscle and elastic fibres, losing itself in the connective tissue substance of the media. In some cases the lymphoid cell predominates, in others only endothelioid cells are present, and these are seen proliferating from the vessel wall. In still other cases some spindle-shaped endothelioids are present, lying parallel to the muscle layers, and surrounded by collagen substance; an appearance suggestive of cicatrisation. Occasionally a small vessel is seen with a few round endothelial cells round it; there is no collagen groundwork and no destruction of muscle or elastic tissue. I take this to be an early stage in the development of the node.

The adventitia is greatly thickened, so much so that it can be detected by the naked eye. It is mainly due to connective tissue and vascular increase. This vascular increase may be very prominent. It is mainly seen next to the media; the vessels are thin-walled, dilated and probably of recent formation. Some of the larger vessels show proliferative changes in their walls, of endothelial nature. This is so pronounced in some cases as to encroach into the lumen of the vessel, thereby giving it an appearance of endarteritis. The characteristic feature in the adventitia is the cellular infiltration; scattered throughout the adventitia are numerous cells, lymphocytes and endothelials, and occasionally plasma cells. This cellular increase is more marked round the vessels, some of these being completely surrounded by a "cuff" of cells.

In some sections we find evidence of the stages by which the medial lesions described above come into existence. As we trace a series of consecutive sections we find at one spot in the adventitia a highly-congested area. There are a few vessels with perivascular cell collections, chiefly consisting of spindle-shaped endothelials; opposite this in the outer layers of the media there is a gap in which the muscle and elastic elements have disappeared, and this is occupied by an extension of collagen substance from the adventitia. A few spindle-shaped endothelials may be arranged along this gutter. As we trace the sections on, we find that a small thin-walled blood-vessel occupies this space; by its side and parallel to it are usually some spindle-shaped endothelial cells ; farther on in the series of sections this vessel leads to a small focal lesion in the media. It is not uncommon to find more than one example of this nature in one section.

In other sections the adventitia is thickened by dense, wavy bands of connective tissue with very few cells. Near the media there is a proliferation of the vasa vasorum, some of the vessels showing endothelial endarteritis. Along the inner parts of the adventitia there are strands of muscle fibres and elastic tissue parallel to the medial layers and of a ragged and diffuse nature ; they are probably the outer medial layers disturbed from their normal relations by the intense vascular invasion.

Orcein-stained sections show an increase in the elastic tissue elements of the intima. This consists of a fairly thick band of elastic fibres running along the intima and occupying its middle third, in the position of the so-called "stripe." As we trace this elastic band round, it almost totally disappears at the intimal "hillock," where degenerative changes appear greatest. In some sections no evidence of this band is present ; in others there are traces of it in the form of fine, short fibrils running across, or rather skirting this area and taking a curved course around it. In places this band seems to be pushed into the intimal elastic lamina by the degenerative process here. It is an interesting fact that, on the other side of the intimal "hillock," no such band is present.

There are some fine elastic fibrils along the inner branch of the intima just external to the endothelium. The ground substance of the intimal "hillock" stains a greyish-pink colour with fuchsin; lateral to this the thickened intima takes on a deep pink along the middle third. However, in many sections there is not much elastic tissue increase. The internal elastic lamina presents different appearances; in some sections it is intact, in others it is broken up, stretched or distorted. It may present merely a series of short elastic fibres, often fenestrated by the collagenous matrix of the intima as it invades the media.

In the inner third of the media we find the elastic fibres separated from each other by well-formed collagenous tissue; the elastic laminæ have in some cases lost their parallel and regular appearance so characteristic in the normal aorta. The elastic fibres may be torn and be present only in short shreds throughout the whole thickness.

In the outer medial layers there is a complete absence of elastic tissue in the areas occupied by the medial lesions; the laminæ are abruptly broken up at the margins of these areas, and only in a few cases do I find traces of elastic fibrils traversing them. In other instances the elastic tissue is pushed aside by these medial foci. Some elastic fibres are seen in the adventitia, next to the media; they are in short shreds, and probably belong to the media mentioned before.

In a few sections stained with Sudan III. and Scarlet Red small globules of fat can be seen scattered about the intima, mostly at its thickest portions. In the deep part of the intimal "hillock" and in the adjacent areas of the media there is a large deposit of fat, consisting of globules of varying sizes; these have fused together in the centre forming a large mass. In places these fatty globules extend deep into the media, occupying spaces between the muscle fibres and pushing them aside, thereby accounting in some measure for the looseness of the muscular cells seen in the hæmatoxylin and eosin sections. Many of the muscle cells themselves contain fat droplets, these being present especially in those cells near the fatty deposit, and this is probably the initial step of the degenerative process; as the fat gradually fills the cell the latter disappears and the fat comes to lie in the intermuscular spaces. The collection of fat is fairly localized, and as we trace it laterally it gradually disappears, as the intimal "hillock" passes into the less thickened portions of the intima.

In other sections a striking appearance is seen. Large, irregular collections of fat are present in the sub-intimal layers of the media; there is little, if any, in the overlying intima. The internal elastic lamina may be unaltered, and course over the fat deposit undisturbed. That such localized collections of fat are present is not to be wondered at; Andrewes,[7] writing on arterial degeneration, says, "Whereas the slighter degrees of fatty changes are usually diffuse, the more severe degrees tend to be patchy in distribution. Such local foci of degeneration are situated remote from the vasa vasorum."

CASE B.—The intima shows the puckering noticed in the other sections. There is much fibrous tissue in the intima; the musculo-elastic layer is present in sections. The elastic tissue of the intima is also increased. The internal elastic lamina is not easy of recognition, making the transition from intima to media difficult to localize.

In the media there is considerable increase of fibrous tissue; the elastic is present as very fine shreds. The muscle fibres are greatly diminished in number. These facts are very striking in orcein sections. What muscle there is is grossly disturbed; the nuclei of the cells appear fragmented and drawn out with their long axes pointing in all directions, instead of the familiar parallel arrangement. In the outer layers of the media there is a focus of cells similar to that described above. The adventitia shows a few perivascular collections of cells.

CASE C.—The intima is slightly increased in thickness. The media in some sections shows increase of intermuscular connective tissue; the muscle cells show somewhat similar changes to the previous case, their nuclei lie scattered irregularly throughout.

The deeper layers of the media are interrupted by the focal lesions previously described. In this case the lesions are very vascular, some of them in a small area contain two or even three thin-walled capillaries; the cells are few. There is much collagenous material around them.

In the adventitia there is fibrous and vascular increase; some of the adventitial vessels exhibit endothelial proliferation showing a well-marked appearance of endarteritis. These vessels have usually a "cuff" of endothelial cells surrounding them. The perivascular cellular increase is very marked; in addition to the two common types of cells found hitherto, *i.e.* endothelioids and lymphocytes, there are here large cells, usually polygonal, occasionally elongated with a more or less central nucleus. The cell takes the eosin fairly deeply, and the nucleus, though taking the hæmatoxylin well, has a loose chromatin structure which makes it appear vacuolated. These cells are larger than plasma cells, and resemble the giant cells seen in the rheumatic nodules in the myocardium etc., except that in no case could I see more than one nucleus.

CASE D.—Here the intima was not much altered. The media showed similar focal lesions as hitherto described. In this case these foci are rather cellular ; a long band of collagenous matrix, extending circularly round the vessel for a considerable way, may only contain a blood vessel and a few endothelial cells round it. This collagenous substance is usually found extending from the adventitia. In orcein sections an increase of the intermuscular matrix is found similar to the previous cases.

The adventitia shows a rich vascularity, and some of the vessels show intimal endothelial proliferation. Round the vessels and scattered throughout the adventitia are numerous cells ; endothelials, monocytes and many plasma cells. These cells were not seen in such numbers in any of the other cases. It is interesting to note that in this case plasma cells are a predominant feature in other organs.

CASE E.—There is some intimal increase. In the media there are again the focal lesions previously described. As in case D, these are not very cellular. The adventitia shows cellular infiltration round the vasa vasorum. Orcein sections show an increase of fibrous tissue in the intima and the media.

COMMENT.—Two apparently different appearances, that in the intima and that in the media, are to be accounted for.

What relation, if any, has the intimal change to the underlying pathological process in the media? Klotz[8] has said: "We believe with Koester that, in as far as the inflammatory processes of the media associated with rheumatism are concerned, the intimal thickening is secondary." Pappenheimer and Von Glahn,[5] on the other hand, regard the intimal changes as a diffuse reaction to infection from the lumen. It is scarcely possible that the character of the intimal increase as described is wholly of the nature of a compensatory process as Klotz suggests. The appearances indicate an inflammatory response of a subacute nature, to an infection from the blood-stream. Whether this response is specific for rheumatic fever it is scarcely possible to say. Pappenheimer and Von Glahn believe it to be so. They describe, what they regard as characteristic, the formation of "palisades" and the presence of Aschoff cells. I have not been fortunate in this last respect. I have seen the "palisade" appearance in the intima, but only in a few sections. Moreover, I have seen typical "palisades" in the intima of the aorta in a case of acute tuberculosis. The intimal change cannot, however, be wholly explained as inflammatory. I suggest that there is at the same time an attempt on the part of the intima to make good the damage done to the underlying media. These I believe to be strongly supported by the increase in fibrous and elastic tissue; especially I refer to the elastic band running through a portion of the intima as described above. I do not know of any condition where elastic increase takes place as a response to an infection. It might be argued that the elastic increase is of the nature of the normal "stripe" seen in the developing arteries. If this were so, one would expect to find this elastic tissue present in all sections and throughout the whole of the intima, whereas, as mentioned before, it is only present at one side of the intimal "hillock." Furthermore, the normal stripe in a subject of thirteen years is never of such thickness and density. This elastic increase corresponds with the "local hyperplastic sclerosis" of Andrewes,[7] which he describes as occurring at points of special stress. The origin of this elastic tissue is a much debated point. Jores[9] believed it to be the product of the internal elastic layers. Evans,[10] in his Goulstonian Lectures, mentions the possibility of the endothelium of the intima being responsible for the elastic production. The appearances in my sections seem to support this view. I have described the arrangement of flattened endothelials lying along the intima, and it is amongst these cells that the elastic band is found. Furthermore, it is difficult to believe that an internal elastic lamina so damaged as that in Case A could possess such vitality as the production of this elastic would entail.

The changes found in the inner layers of the media can be explained by the impairment of the nutrition of these medial layers, due to the thickening of the intima. Hence, here also in the media we find the fatty change most pronounced, the highly-specialized muscle cells bear the deprivation of nourishment very badly. The perivascular lesions found in the outer third of the media correspond with the findings of Pappenheimer and Von Glahn[5] in the aorta and Kugel and Epstein[11] in the pulmonary artery. One may hesitate to define these lesions as typical Aschoff bodies, but there can be no doubt that the type of reaction here present is essentially that found in rheumatic manifestations elsewhere. The response is similar, only differing in degree and modified by the histology of the part in which it occurs.

Cases C, D, and E have been included to show how the appearance may be modified in degree, and also by the age of the lesion, these losing their cellular characters and finally cicatrising. Probably in many cases they leave little evidence of their former presence.

In Case B the findings might have been accounted for by the age of the patient and the renal changes present, but for the fact of the well - marked inflammatory foci in the media. We can exclude syphilis both macroscopically and microscopically: periarteritis nodosa never occurs in the aorta, and can therefore be disregarded. Moreover, the microscopical appearance of these focal lesions in Case E is similar to that found in the other cases, and may, therefore, be placed with them. This case emphasizes the fact that a rheumatic infection which has been dormant for many years may, even at the age of sixty, flare up into activity. The pathological features in the adventitia are to be expected if one recalls that through this the infective agent, virus or toxin, has to pass to reach the outer medial layers. The response is also characteristic of the rheumatic infection, being more generalized and diffuse owing to the rich vascularity of this part. Recently Barnard[12] described a case of rheumatic periaortitis; the appearance of the adventitia in Cases C and D might well be the initial stage of such a condition.

### SUMMARY.

1. Five cases of rheumatic fever have been examined histologically.

2. In all of them lesions of a distinctive character have been found in the aortic wall.

3. These consist of areas of subacute inflammation around a small vessel, of a nature similar to those found in the pericardium, myocardium and other tissues in cases of rheumatic fever.

I should like to thank Dr. A. L. Taylor for allowing me to make use of specimens and for his kind help, also Dr. Carey Coombs for much encouragement, without which this would never have been attempted.

### REFERENCES.

[1] Allbutt, *Diseases of the Arteries and Angina Pectoris*, 1915 ed., pp. 148 *et seq.*

[2] Klotz, *Trans. of Assoc. of Amer. Phys.*, 1912, xxvii., 181.

[3] Coombs, *Quart. J. Med.*, 1908, ii., 27.

[4] MacCallum, *Bull. Johns Hopkins Hosp.*, 1924, xxxv., 329.

[5] Pappenheimer and Von Glahn, *Jour. Med. Res.*, 1924, xliv., 489; *Amer. Jour. of Path.*, 1926, ii., 235; *ibid.*, 1927, iii., 583.

[6] Coombs, *Jour. Path. and Bact.*, 1911, xv., 489.

[7] Andrewes, *Report on Arterial Degeneration*, 1912.

[8] Klotz, *Jour. of Path. and Bact.*, 1913, xviii., 259.

[9] Jores, *Ziegler's Beitr.*, 1898. 24458.

[10] Evans, *Goulstonian Lectures*, 1923.

[11] Kugel and Epstein, *Arch. of Path.*, 1928, vi.

[12] Barnard, *Jour. of Path. and Bact.*, 1929, xxxii., 95.

## Figures and Tables

**Case A. f1:**
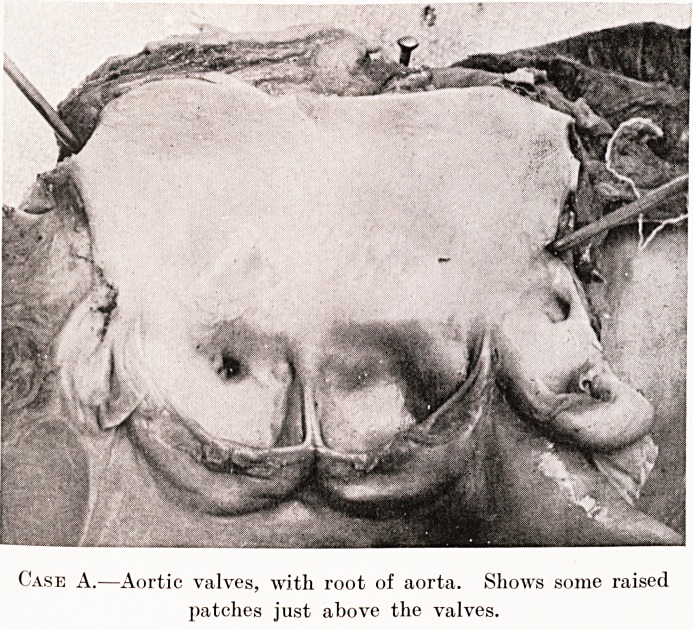


**Case A. f2:**
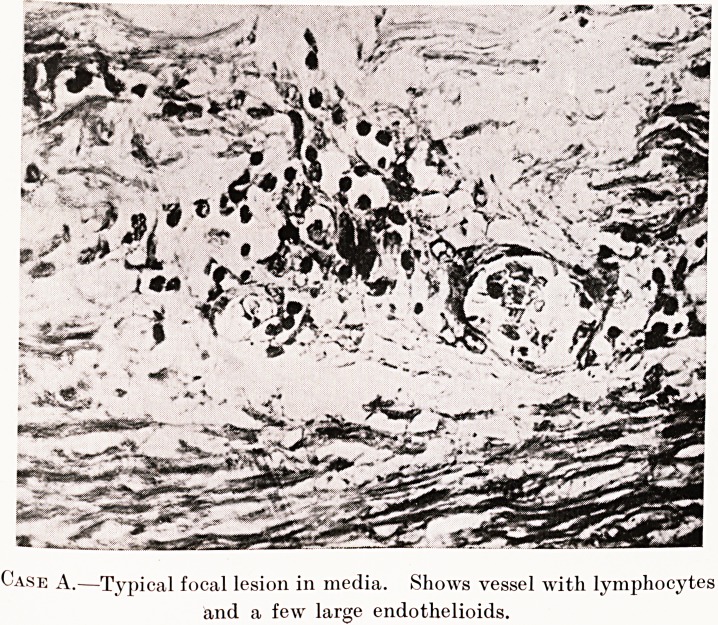


**Case A. f3:**
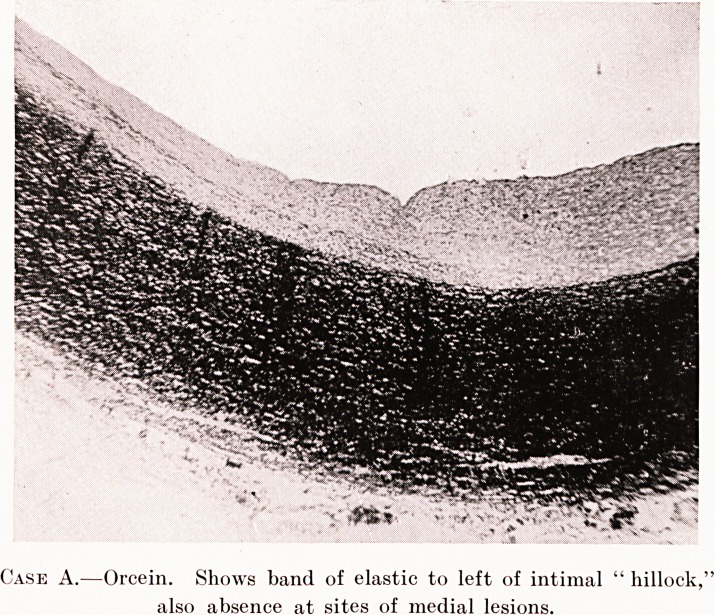


**Case A. f4:**